# Beta-agonist stimulation ameliorates the phenotype of spinal and bulbar muscular atrophy mice and patient-derived myotubes

**DOI:** 10.1038/srep41046

**Published:** 2017-01-24

**Authors:** Carmelo Milioto, Adriana Malena, Eleonora Maino, Maria J. Polanco, Caterina Marchioretti, Doriana Borgia, Marcelo Gomes Pereira, Bert Blaauw, Andrew P. Lieberman, Roberta Venturini, Mario Plebani, Fabio Sambataro, Lodovica Vergani, Elena Pegoraro, Gianni Sorarù, Maria Pennuto

**Affiliations:** 1 Dulbecco Telethon Institute, Centre for Integrative Biology, University of Trento, 38123 Trento, Italy; 2 Department of Neuroscience and Brain Technologies, Istituto Italiano di Tecnologia, 16163 Genova, Italy; 3 Dipartimento di Medicina Sperimentale, University of Genova, 16100 Genova, Italy; 4 Department of Neurosciences, University of Padova, 35100 Padova, Italy; 5 Venetian Institute of Molecular Medicine, Department of Biomedical Science, University of Padova, 35100 Padova, Italy; 6 Department of Pathology, University of Michigan Medical School, Ann Arbor, MI 48109, USA; 7 Department of Laboratory Medicine, University Hospital of Padova, 35100 Padova, Italy; 8 Department of Experimental & Clinical Medical Sciences (DISM), University of Udine, 33100 Udine, Italy

## Abstract

Spinal and bulbar muscular atrophy (SBMA) is a neuromuscular disease characterized by the loss of lower motor neurons. SBMA is caused by expansions of a polyglutamine tract in the gene coding for androgen receptor (AR). Expression of polyglutamine-expanded AR causes damage to motor neurons and skeletal muscle cells. Here we investigated the effect of β-agonist stimulation in SBMA myotube cells derived from mice and patients, and in knock-in mice. We show that treatment of myotubes expressing polyglutamine-expanded AR with the β-agonist clenbuterol increases their size. Clenbuterol activated the phosphatidylinositol-3-kinase (PI3K)/Akt/mechanistic target of rapamycin (mTOR) pathway and decreased the accumulation of polyglutamine-expanded AR. Treatment of SBMA knock-in mice with clenbuterol, which was started at disease onset, ameliorated motor function and extended survival. Clenbuterol improved muscle pathology, attenuated the glycolytic-to-oxidative metabolic alterations occurring in SBMA muscles and induced hypertrophy of both glycolytic and oxidative fibers. These results indicate that β-agonist stimulation is a novel therapeutic strategy for SBMA.

Spinal and bulbar muscular atrophy (SBMA), also known as Kennedy’s disease, is an X-linked neuromuscular disorder characterized by the selective loss of motor neurons from the brainstem and spinal cord^1^. Patients develop progressive atrophy of skeletal muscle, which results in weakness and can lead to the use of canes and wheelchairs in later stages of the disease. SBMA is caused by expansions of a CAG trinucleotide repeat, encoding glutamine, in the androgen receptor (AR) gene^2^. Polyglutamine expansion in different genes is responsible for other neurological conditions, such as Huntington’s disease, dentatorubral-pallidoluysian atrophy, and spinocerebellar ataxia (SCA) type 1, 2, 3, 6, 7, and 17^3^,^4^. Polyglutamine expansion in the AR confers a partial loss of protein function leading to androgen insensitivity, which in some SBMA patients manifests with non-neurological symptoms^5^. In addition, polyglutamine expansion in the AR confers a toxic gain of function that is triggered by the binding of polyglutamine-expanded AR to its natural ligands, testosterone and dihydrotestosterone (DHT)^6^. Hence, SBMA is induced by androgens and consequently manifests primarily in males. Females show only subclinical disease manifestations even when homozygous for the mutation^7^. The sex-specificity of SBMA is well recapitulated in fly and mouse models of disease^8^. Flies develop features of neurodegeneration when grown in food containing testosterone^9^,^10^. Transgenic and knock-in male mice develop disease manifestations, whereas females are not affected or show milder phenotypes compared to males depending on the level of expression of mutant AR^11^,^12^,^13^. Although this evidence supports the use of anti-androgens for SBMA, and clinical trials using this strategy have given promising results^14^,^15^, the use of these drugs is limited by their side-effects.

The development of a therapy for SBMA and for other motor neuron diseases is difficult as motor neurons are poorly accessible pharmacologic targets. An alternative approach consists in targeting peripheral tissues that play key roles in disease pathogenesis, such as skeletal muscle. Recent evidence supports the concept that polyglutamine expansion in the AR causes muscle atrophy through both non-cell-autonomous and cell-autonomous mechanisms^16^. Expression of polyglutamine-expanded AR in skeletal muscle is necessary for development of symptoms in transgenic mice^17^. Moreover, antisense oligonucleotide-mediated inhibition of AR expression in peripheral tissues, including skeletal muscle, attenuated disease manifestations in knock-in SBMA mice^18^. Mechanistically, polyglutamine-expanded AR causes an androgen-dependent impairment of myogenesis in SBMA patients^19^, and alters muscle metabolism^20^. Overall, this evidence supports a primary role for muscle in disease pathogenesis and candidates this tissue as a valuable target for therapy development.

Stimulation of β-adrenergic signaling with β-adrenoceptor agonists (β-agonists), such as clenbuterol, is emerging as an effective strategy to counteract skeletal muscle wasting and atrophy^21^,^22^. Clenbuterol has been shown to exert beneficial effects in animal models of muscular dystrophy^23^, amyotrophic lateral sclerosis (ALS)^24^, cancer cachexia^25^, and denervation^26^. A large number of clinical trials with β-agonists, including clenbuterol, has been conducted in patients suffering from neuromuscular disorders^27^,^28^. Importantly, results of a one-year pilot study with clenbuterol in SBMA patients were consistent with beta-adrenoceptor stimulation efficacy in improving motor function with no relevant adverse events^29^. β-agonists bind to adrenoceptors, which are G protein-coupled receptors that induce muscle hypertrophy mainly through activation of two cellular pathways, namely the PI3K/Akt and adenylyl cyclase (AC)/protein kinase A (PKA) pathways. The PI3K/Akt pathway plays a key role in skeletal muscle homeostasis^30^. Activation of Akt promotes muscle hypertrophy by regulating the function of several downstream effectors, such as mTOR. mTOR promotes new protein synthesis by targeting the eukaryotic translation initiation factor 4E binding protein 1 (4EBP1) and S6 kinase 1 (S6K1), which in turn phosphorylates S6. In addition to exerting anabolic effects on skeletal muscle, the activation of the PI3K/Akt pathway may have specific effects on SBMA muscle by promoting phosphorylation-mediated polyglutamine-expanded AR degradation^31^,^32^,^33^,^34^.

Here, we carried out a preclinical study to assess the efficacy and identify the mechanism of action of clenbuterol in SBMA muscle cells and knock-in mice. We found that treatment with clenbuterol reduces myotube atrophy and ameliorates the phenotype of SBMA mice, thereby supporting the use of β-agonists as a promising therapeutic strategy for SBMA patients.

## Materials and Methods

### Animals and treatments

Animal care and experimental procedures were conducted in accordance with the University of Trento ethics committee and were approved by the Italian Ministry of Health. Generation and genotyping of knock-in AR113Q mice were previously described^35^. Only male mice were used in this study. Mice were housed in filtered cages in a temperature-controlled room with a 12-hour light/12-hour dark cycle with *ad libitum* access to water and food, and they were fed a standard diet [Special Diet Services - 811900 VRF1(P)]. Ninety-day-old mice were randomly assigned to three groups and were treated with vehicle (water), 1 mg/kg, and 2 mg/kg clenbuterol hydrochloride ≥95% (Sigma-Aldrich). Treatment was carried out 3 consecutive days per week to avoid desensitization of adrenoceptors, and it was administrated by oral gavage. Treatment of the animals was started at disease onset (13 weeks of age) and was conducted for 15 weeks. The operator was blind for genotype and treatment (a color code was used to identify the three groups). Each mouse was weighted before treatment administration. Motor coordination was measured by rotarod analysis (Ugo Basile Instruments): mice received a weekly session that included three test trials at 21 rpm speed for a maximum period of 600 seconds each, with a recovery time of 300 seconds between each test. Mice were trained the week before starting the test. The average of recordings for each mouse was used to analyze rotarod performance. For grip strength analysis of muscle force, a grip strength meter (Ugo Basile Instruments) was used to measure forelimb grip strength. The grip strength meter was positioned horizontally, and the mice were held by the tail and lowered toward the apparatus. Mice were allowed to grasp the smooth metal triangular pull bar with their forelimbs only and then were pulled backward. The force applied to the bar, measured at the moment in which the grasp was released, was recorded as the peak tension. Mice received a weekly session which included three test trials, and the highest recording for each mouse was used to analyze muscle force production. To measure muscle force in living animals, the contractile performance of gastrocnemius muscle was measured in living animals as previously described^36^. Briefly, anesthetized mice were placed on a thermostatically controlled table, keeping the knee stationary, and the foot firmly fixed to a footplate, which was connected to the shaft of the motor of a muscle-lever system (305B, Aurora Scientific). Contraction was elicited by electrical stimulation of sciatic nerve. Teflon-coated seven-stranded steel wires (AS 632, Cooner Sales) were implanted with sutures on either side of the sciatic nerve proximal to the knee before its branching. At the distal ends of the two wires, the insulation was removed, and the proximal ends were connected to a stimulator (S88, Grass). To avoid recruitment of the dorsal flexor muscles, the common peroneal nerve was cut.

### Cell cultures

Generation of stable C2C12 cell clones was carried out by lentiviral transduction. Full length AR24Q and AR100Q cDNAs were cloned into the lentiviral vector #945.PCCL.sin.cPPT.SV40ployA.eGFP.minCMV.hPGK.deltaLNGFR.Wpre, in which transgene expression is driven by the human phosphoglycerate kinase (PGK) promoter, and enhanced green fluorescent protein (EGFP) expression is driven by the cytomegalovirus promoter. The production of VSV-pseudotyped third-generation lentiviruses was performed as previously described^37^. C2C12 cells were infected at 10 multiplicity of infection. After 24 hours, the medium was replaced with fresh medium. After 6 weeks post-infection, transduced cell lines were analyzed and sorted by flow cytometry with the BD FACS Aria II™ cell sorter (Becton Dickinson, USA), based on EGFP expression. C2C12 cells stably expressing AR24Q and AR100Q were maintained in growth medium (GM) composed of Dulbecco’s modified Eagle’s medium (DMEM, Life Technologies) supplemented with 10% fetal bovine serum (FBS, Life Technologies), 1% penicillin/streptomycin, and 1% glutamine (Life Technologies). C2C12 cells were used at passages 11–17. To induce myoblast differentiation to myotubes, the C2C12 cells were seeded at a density of 7000 cells/cm^2^. Two days after seeding (day 0), GM was changed, and the cells were cultured in differentiation medium (DM) composed of DMEM supplemented with 2% horse serum (HS, Life Technologies), 1% penicillin/streptomycin, and 1% glutamine for 14 days. Every two days DM was changed and the cells were treated starting from day 0 with ethanol or DHT (10 nM, Sigma) together with either vehicle (water) or clenbuterol (10 μM, Sigma). Before lysis, the cells were incubated for 2 hours in phosphate-buffered saline (PBS, Life Technologies) to promote starvation-induced cell synchronization. Then, the cells were treated for 30 minutes with vehicle, DHT (10 nM), and clenbuterol (10 μM) before processing for further analysis.

Anonymized control and patient biopsy sample collection was approved by the ethics committee of the University of Padova (Italy). Written informed consent was obtained from each patient. Confidentiality was guaranteed by assigning a study code to each patient. All patients who underwent muscle biopsy were clinically affected and showed weakness and/or fasciculation and/or muscle atrophy. Myopathic changes together with neurogenic atrophy were observed in muscle biopsies. Primary myoblast cells were extracted from quadriceps femoralis muscle biopsies taken from five SBMA patients and four healthy age-matched male control subjects. Cells were cultured in Ham’s F14 medium (Biowest) plus 30% fetal bovine serum (FBS, Gibco, Invitrogen) and 10 mg/ml insulin (Sigma). Cells at 70% confluence were differentiated by lowering FBS to 2%. All the proliferating and differentiating cell lines were cultured in medium containing either ethanol or DHT (10 nM) with or without Clenbuterol (10 μM).

### Morphological analysis

Bright-field images of C2C12 myotubes were taken using a Leica DM IL LED microscope equipped with a standard camera. Average myotube width was measured using Image-Pro Premier 9.1 software (Media Cybernetics). Briefly, once the borders of each myotube were drawn by two lines, the software measured the average width between these two lines. Then, 10/12 images (up to 50 myotubes/condition) randomly taken for each different condition were analyzed for each independent experiment to assess myotube width. Bright-field images of living human myotubes (passage number 4-to-8) were collected using a Zeiss IM35 microscope equipped with a standard camera. Fusion index was quantified by counting the nuclei in at least 20 myotubes per cell line using contrast phase microscopy, as we have previously described^19^. Fusion index was calculated as the average number of nuclei per myotube obtained by dividing the number of nuclei in myotubes by the total number of myotubes. Average myotube width at 10 days of differentiation was measured using ImageJ ProPlus 6 software as described for C2C12 cells.

Immunofluorescence analyses were performed in myotubes fixed in 4% paraformaldehyde, permeabilized with 0.2% Triton X-100 and incubated for 30 min in 2% BSA in PBS. The AR antibody (N-20, Santa Cruz SC-816) was diluted in PBS plus 2% BSA and incubated overnight at 4 °C. Secondary Alexa 488 antibody (A11008, ThermoFisher Scientific) was incubated for 1 h at room temperature. Samples, mounted in Vectashield mounting medium with DAPI (4′-6-diamidino-2-phenylindole, H-1200, Vector Laboratories), were analyzed with an Olympus BX60 fluorescence microscope.

### Histological Analysis

For tissue analysis, mice were sacrificed after 15 weeks of treatment. Two hours after the last treatment, mice were euthanized by isofluorane inhalation. After euthanasia, tissues were weighted and flash-frozen in isopentane precooled in liquid nitrogen, then stored at −80 °C until further processing. Gastrocnemius muscles were embedded in optimal cutting temperature (OCT) compound (Tissue Tek, Sakura, Torrance, CA), and cross sections (10 μm-thick) were cut with a cryostat (CM1850 UV, Leica Microsystem). Cryosections of gastrocnemius were stained for nicotinamide adenine dinucleotide (NADH). For NADH, sections were air-dried and incubated in NADH solution [0.2 M TRIS-HCL, Nitrotetrazolium Blue chloride (Sigma), β-nicotinamide adenine dinucleotide, (Sigma)] at 37 °C for 40 minutes. Sections were briefly washed in 30%, 60%, 90%, 60%, and 30% acetone solutions. Sections were then washed in water, dried, and mounted with aqueous mounting medium [Gelatin (Serva), sodium azide (Merck), and glycerin]. Images were taken using a Nikon Eclipse 90i upright microscope, the analyses were performed using ImageJ software, and pixel number was converted to μm^2^. The number of fibers and mean cross-sectional area (CSA) distribution were evaluated by a blinded investigator from 3–5 randomly selected areas of 3 sections of each muscle for each genotype.

### Biochemical Analysis

Cells were washed in ice-cold PBS and lysed in ice-cold RIPA buffer (150 mM NaCl, 6 mM Na_2_HPO_4_, 4 mM NaH_2_PO_4_, 2 mM EDTA pH8, 1% Na-deoxycholate, 0.5% TritonX-100, 0.1% SDS) plus protease (Roche Diagnostics) and phosphatase (Sigma) inhibitor cocktails. The lysates were collected, sonicated, and cleared by centrifugation at 4 °C at 13000 rpm. Gastrocnemius muscles were collected immediately after euthanasia and then flash-frozen in isopentane precooled in liquid nitrogen and stored at −80 °C until further processing. Frozen muscles were mechanically pulverized and homogenized in lysis buffer [20 mM HEPES, 5 mM EGTA, 2% sodium dodecyl sulfate (SDS)] containing protease (Roche Diagnostics) and phosphatase (Sigma) inhibitor cocktails, boiled for 3 minutes, and centrifuged for 20 minutes at 13,000 rpm at room temperature. Soluble fractions were collected and protein concentration was determined using the bicinchoninic acid (BCA) assay method (Thermo Scientific). For Western blotting, lysates were denatured at 95 °C in 5X sample buffer (0.5 M Tris-HCL, pH = 6.8, 10% SDS, 25% glycerol, 0.5% bromophenol blue, 0.5% β-mercaptoethanol, deionized water) and separated on SDS-polyacrylamide gels (SDS-PAGE). Gels were electrotransferred to nitrocellulose membranes (Thermo Scientific; pore size = 0.45 μm). Membranes were blocked in either 5% dry milk or 5% BSA in 0.02 M Tris/HCl pH = 7.5, 137 mM NaCl, and 0.1% (v/v) Tween-20 for 1 h at room temperature. The following antibodies were used: Cell Signaling Technology: phospho-Ser473-Akt (9271 S), total Akt (9272), phospho-Ser133-CREB (9198), total CREB (9197), phospho-Ser240/244-S6 (2215), total S6 (2317); AR (Santa Cruz SC-816); Calnexin (Enzo Life Sciences, ADI-SPA 860), β-tubulin (Sigma, T7816), and β-actin (Millepore, MAB1501). Signal intensities were quantified by ImageQuant LAS 4000 mini (GE Healthcare BioSciences) or using LI-COR Odyssey Infrared Imaging System (LI-COR Biosciences). Quantifications were performed using ImageJ software. Alternatively, after incubation with secondary HRP-conjugated antibodies, signals were visualized by chemiluminescence (GE HealthCare), and integrated optical density of each band was calculated with commercial software (Gel Pro Analyzer). For analysis of serum creatine-kinase (CK) levels, a blood sample was collected immediately after euthanasia to obtain serum by ultracentrifugation. Serum was then stored at −80 °C until further processing. Serum creatine-kinase (CK) levels were measured by a spectrophotometric assay (Roche Diagnostics, Mannheim).

### Statistical analysis

All data are presented as mean ± standard error of the mean (SEM). Data normality was tested using Kolmogorov-Smirnov goodness-of-fit test. For normal data, two-sample Student’s t-test and two way ANOVA were used when two or more than two groups were available, respectively. One way ANOVA was followed up by Student-Newman-Keuls (SNK) post-hoc tests. For non-parametric data, we used Kruskal-Wallis ANOVA test followed by multiple comparisons Kruskal-Wallis post-hoc tests (Dunn’s test). For longitudinal behavioral analyses, linear mixed-effect model with the behavioral readouts (time at the rotarod test and strength at the grip strength test, respectively) as a dependent variable, mouse as a random-effect, and time, group and their interaction as a fixed-effect using the lme4 package in R^38^. To identify the time when behavioral differences become significant, we used one-way ANOVA with planned comparisons [(clenbuterol ≥ vehicle within AR113Q mice), and (vehicle AR113Q ≥ vehicle control mice)] at each time point. Comparisons of survival curves between treatment and control-vehicle groups were performed using Log-Rank test. Statistical significance threshold was set at p < 0.05. All statistical analyses were performed using STATISTICA 8 (StatSoft, Inc.), R 3.3.1 (http://CRAN.R-project.org), and GraphPad Prism 5 (GraphPad Software, Inc.).

## Results

### Clenbuterol attenuates the detrimental effects of androgens in C2C12 myotubes expressing polyglutamine-expanded AR

**Figure 1:**
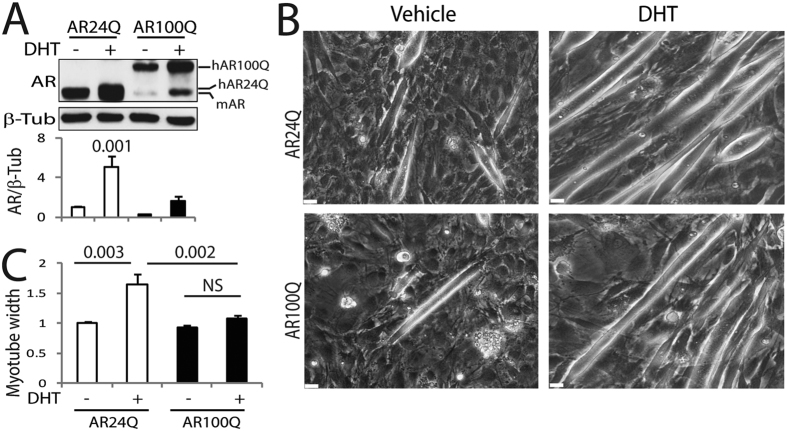
Androgen treatment of C2C12 myotubes expressing polyglutamine-expanded AR induces atrophy. (**A**) Western blotting analysis of C2C12 cells stably expressing human non-expanded AR (AR24Q) and polyglutamine-expanded AR (AR100Q) and differentiated to myotubes in the presence of vehicle and DHT (10 nM) for 14 DIV. hAR, human AR; mAR, endogenous mouse AR. AR was detected with a specific antibody, and beta-tubulin (β-Tub) was used as loading control. Graph, mean ± SEM, n = 5 independent experiments. Two-way ANOVA + SNK. (**B**) Representative bright-field images of the C2C12 myotubes cultured for 14 DIV in the presence and absence of DHT (10 nM). Bar, 25 μm. (**C**) Myotube width analysis of the C2C12 myotubes expressing AR24Q and AR100Q and treated with either vehicle or DHT (10 nM) for 14 DIV. Graph, mean ± SEM, n = 3 independent experiments. Two-way ANOVA + SNK. NS, non-significant.

**Figure 2:**
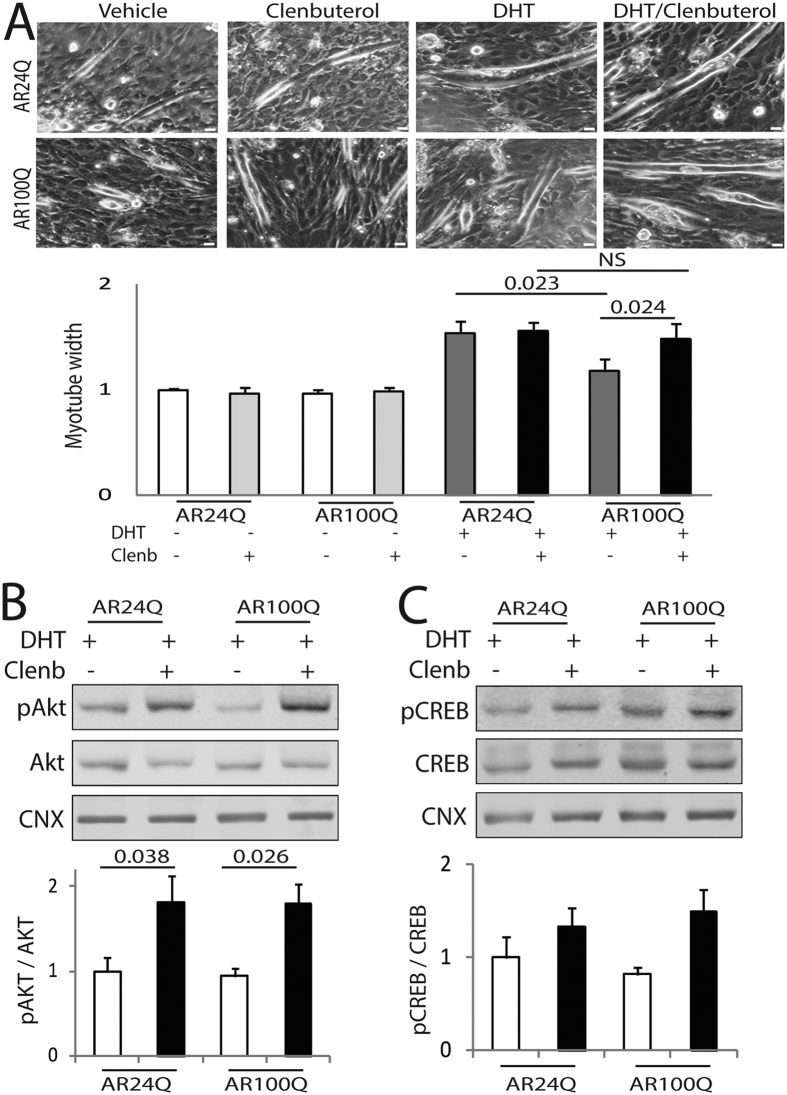
Clenbuterol mitigates the atrophy induced by polyglutamine-expanded AR in C2C12 myotubes. (**A**) Upper panels, representative bright-field images of C2C12 myotubes expressing AR24Q and AR100Q treated with vehicle, DHT (10 nM), and clenbuterol (clenb, 10 μM) for 14 DIV. Bottom panel, myotube width analysis. Graph, mean ± SEM, n = 3 independent experiments. Two-way ANOVA + SNK. NS, non-significant. Bar, 25 μm. (**B**,**C**) Western blotting analysis of phosphorylated and total Akt (**B**) and CREB (**C**) in C2C12 myotubes expressing AR24Q and AR100Q and cultured as in (**A**). Phosphorylated and total Akt and CREB were detected with specific antibodies, and calnexin (CNX) was used as loading control. Graph, mean ± SEM, n = 6 (**B**) and 5 (**C**) independent experiments. Two-way ANOVA + SNK.

To test the efficacy of clenbuterol in SBMA myotubes, we generated a novel muscle cell model of SBMA by transducing C2C12 myoblast cells with bicistronic lentiviral vectors expressing soluble EGFP and either human non-expanded AR with 24 glutamine residues (AR24Q) or polyglutamine-expanded AR with 100 glutamine residues (AR100Q)^39^,^40^. EGFP-positive cells were recovered by fluorescence-activated cell sorting (FACS). Myoblast cells were differentiated to myotubes for 14 days in culture (DIV) in the presence and absence of DHT, and AR expression was analyzed by Western blotting (Fig. 1A). The levels of expression of AR24Q were 3-fold higher than those of AR100Q, and AR levels were augmented upon DHT treatment, as expected^32^. We have previously shown that DHT treatment of primary myotubes derived from normal subjects increases myotube size, a trophic effect of androgens that is lost in myotubes derived from SBMA patients^19^. Therefore, we asked whether DHT treatment has the same effect in this new C2C12 cell model of SBMA (Fig. 1B and C). Treatment with DHT significantly increased the myotube size of AR24Q-expressing cells by 1.6-fold, and had no effect on the AR100Q-expressing cells, thereby indicating that the SBMA C2C12 myotubes recapitulate features observed in cultured myotubes derived from SBMA patients. Therefore, we used this new SBMA muscle cell model to assess the effect of clenbuterol on SBMA myotube size (Fig. 2A). In the absence of DHT, clenbuterol did not affect C2C12 myotube size. In the presence of androgens, clenbuterol significantly increased the size of myotubes expressing polyglutamine-expanded AR by 1.25-fold, indicating that clenbuterol promotes hypertrophy of SBMA myotube cells. To determine whether clenbuterol activates the PI3K/Akt pathway, we measured Akt phosphorylation at serine 473^41^. In the presence of androgens, clenbuterol significantly increased the phosphorylation of Akt by 1.8-fold in control and SBMA myotubes (Fig. 2B). Moreover, clenbuterol increased the phosphorylation of S6 in SBMA myotubes, even if this effect was not significant (Supplementary Figure 1). To determine whether clenbuterol activates the AC/PKA pathway, we measured cAMP response element-binding protein (CREB) phosphorylation at serine 133^42^. Clenbuterol increased the phosphorylation of CREB (although not significantly) by 1.3- and 1.8-fold in control and SBMA myotubes, respectively (Fig. 2C). Taken together, these results indicate that clenbuterol activates the PI3K/Akt pathway and increases the size of SBMA myotubes.

### Clenbuterol treatment ameliorates the phenotype of knock-in SBMA mice

**Figure 3:**
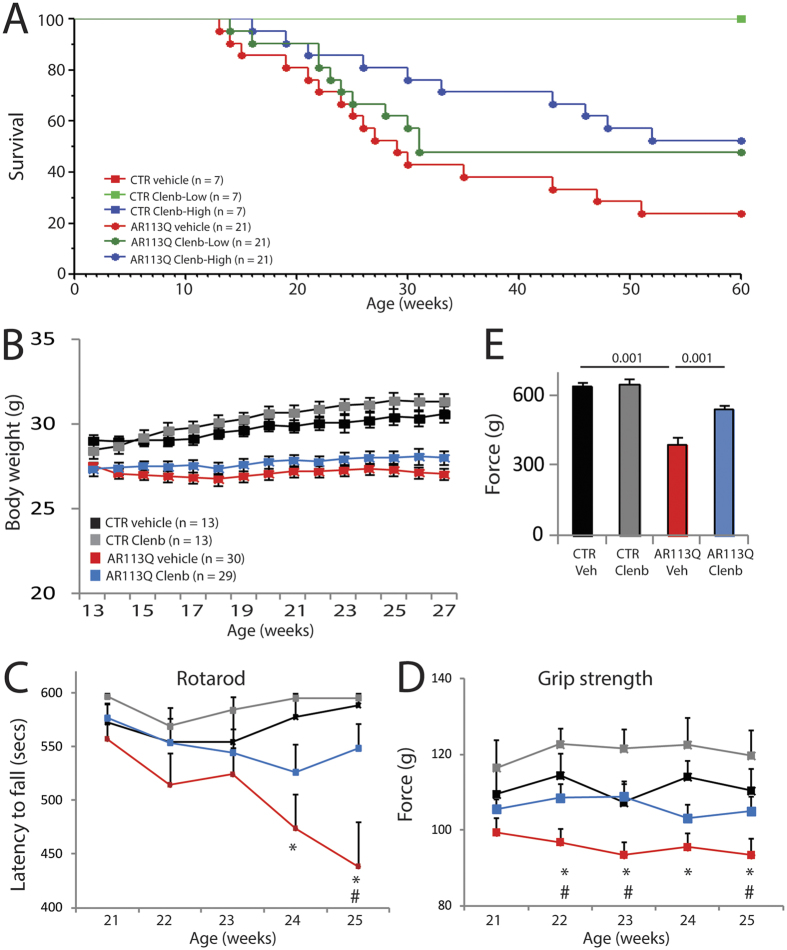
Clenbuterol ameliorates the life span and motor function of SBMA knock-in mice. (**A**) Kaplan-Meier analysis of survival of control (CTR, wild type, square) and AR113Q (circle) mice treated with vehicle (red) and clenbuterol (Clenb, 1 mg/kg, green; 2 mg/kg, blue). (**B**) Body weight analysis of CTR and AR113Q mice treated with vehicle (CTR, black; AR113Q, red) and clenbuterol (2 mg/kg; CTR, grey; AR113Q, blue). Graph, mean ± SEM. (**C**) Rotarod analysis of motor coordination in CTR and AR113Q mice, n = as in (**B**). Graph, mean ± SEM. Week 24: *p = 0.015 CTR-vehicle vs AR113Q-vehicle. Week 25: *p = 0.002 CTR-vehicle vs AR113Q-vehicle, ^#^p = 0.005 AR113Q-vehicle vs AR113Q-clenbuterol. (**D**) Grip strength analysis of CTR and AR113Q mice treated as in (**B**). Graph, mean ± SEM. Week 22: *p = 0.007 CTR-vehicle vs AR113Q-vehicle, ^#^p = 0.026 AR113Q-vehicle vs AR113Q-clenbuterol. Week 23: *p = 0.028 CTR-vehicle vs AR113Q-vehicle, ^#^p = 0.003 AR113Q-vehicle vs AR113Q-clenbuterol. Week 24: *p = 0.007 CTR-vehicle vs AR113Q-vehicle. Week 25: *p = 0.03 CTR-vehicle vs AR113Q-vehicle, ^#^p = 0.06 AR113Q-vehicle vs AR113Q-clenbuterol. (**E**) *In vivo* force generation of gastrocnemius muscle measured in live 180-day-old AR113Q and CTR mice treated as in (**B**). Graph, mean ± SEM, n = 4 mice.

Based on these results, we performed a preclinical study to assess whether clenbuterol modifies the toxicity of polyglutamine-expanded AR *in vivo*. To this aim, we used SBMA knock-in mice. In these mice, the exon 1 of the *AR* gene was replaced with the human exon 1 with a polyglutamine tract length of 113 glutamine residues (AR113Q)^35^. As control, we used wild type sibling mice. AR113Q mice develop muscle atrophy starting around 8–12 weeks of age, show reduced muscle force and altered motor function, and about 50% of them die prematurely from urinary tract obstructions by 30 weeks of age^13^,^43^. Treatment of the mice with clenbuterol was performed by oral gavage 3 days/week to avoid receptor desensitization, using a “low” dose (1 mg/kg) and a “high” dose (2 mg/kg) of clenbuterol. The operator was blind for genotype and treatment. Treatment was started at disease onset (13 weeks of age), which is the time at which the AR113Q mice show reduced body weight and an altered pattern of gene expression^20^, and was ended at 27 weeks of age. Clenbuterol did not modify the survival of control mice (Fig. 3A). Treatment with the low-dose clenbuterol slightly and not significantly extended the survival of AR113Q mice, whereas treatment with the high-dose clenbuterol significantly (χ^2^ Log-Rank = 4.738, *P* = 0.0295) extended the life span of AR113Q mice by 30 weeks of age. Therefore, we focused further analysis of mouse phenotype on the high dose-treated group. AR113Q mice have reduced body weight compared to age-matched control mice, and treatment with clenbuterol did not modify the body weight of both AR113Q and control mice (Fig. 3B). Clenbuterol significantly ameliorated motor coordination of AR113Q mice assessed by rotarod task (Fig. 3C, Supplementary Figure 2A), increased muscle force measured by grip strength (Fig. 3D, Supplementary Figure 2B), and enhanced tetanic force production by 1.4-fold measured in the gastrocnemius muscle of living animals (Fig. 3E, Supplementary Figure 2C). These results indicate that chronic β-agonist stimulation ameliorates the phenotype of SBMA knock-in mice.

### Clenbuterol reduces muscle pathology in knock-in SBMA mice

**Figure 4:**
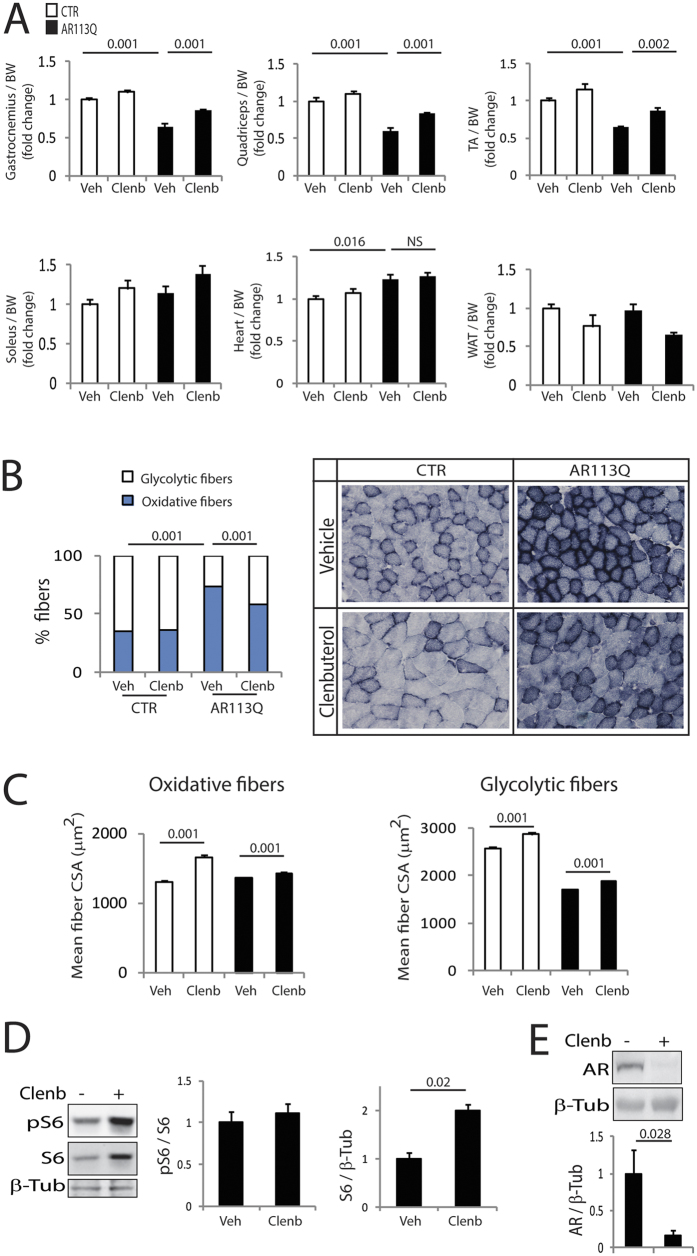
Clenbuterol reduces muscle atrophy and metabolic alterations in SBMA knock-in mice. (**A**) Analysis of tissue weight normalized to body weight (BW) of 180-day-old control (CTR, wild type) and AR113Q mice treated with vehicle (Veh) and clenbuterol (Clenb, 2 mg/kg). TA, tibialis anterior; WAT, white adipose tissue. Graph, mean ± SEM, n = 6 CTR-vehicle, 6 CTR-clenbuterol, 8 AR113Q-vehicle, and 6 AR113Q-clenbuterol mice. Two-way ANOVA + SNK. (**B**) NADH staining of gastrocnemius muscle from 180-day-old AR113Q and CTR mice treated as in (**A**). Graph, mean ± SEM, n mice = 5 CTR-vehicle, 5 CTR-clenbuterol, 5 AR113Q-vehicle, and 5 AR113Q-clenbuterol, n fibers = 1222 CTR-vehicle, 1034 CTR-clenbuterol, 1810 AR113Q-vehicle, and 1650 AR113Q-clenbuterol. Right, representative images. Two-way ANOVA + SNK. (**C**) Analysis of the mean oxidative and glycolytic myofiber CSA in the gastrocnemius muscle of 180-day-old AR113Q and CTR mice treated as in (**A**). Graph, mean ± SEM, n as in (**B**). Two-way ANOVA + SNK. (**D**) Western blotting analysis of phosphorylated and total S6 in the gastrocnemius muscle of 180-day-old AR113Q mice treated as in (**A**). S6 was detected with a specific antibody, and beta-tubulin (β-Tub) was used as loading control. Graph, mean ± SEM, n = 6 mice for each group. Student’s’ t Test. (**E**) Western blotting analysis of polyglutamine-expanded AR levels in the gastrocnemius muscle of 180-day-old AR113Q mice treated as in (**A**). AR was detected with a specific antibody, and beta-tubulin (β-Tub) was used as loading control. Graph, mean ± SEM, n = 7 mice for each group. Student’s t-test.

Next, we asked whether the improvement of motor function induced by clenbuterol was associated with an amelioration of muscle pathology. In knock-in SBMA mice, the expression of polyglutamine-expanded AR induces atrophy of skeletal muscles composed of both type I slow-oxidative and type II fast-glycolytic fibers, namely gastrocnemius, quadriceps, and tibialis anterior (TA), but not muscles mainly composed of type I slow-oxidative fibers, namely soleus^20^. Clenbuterol treatment increased the mass of gastrocnemius, quadriceps and TA muscles by 1.4-fold compared to the vehicle-treated AR113Q mice (Fig. 4A). Moreover, clenbuterol slightly and not significantly increased the mass of soleus muscle by 1.25-fold. β-agonists may cause hypertrophy of cardiac muscle. The mass of heart was greater in the AR113Q mice compared to control mice, and was not altered by clenbuterol treatment. Moreover, we did not detect any heart gross abnormalities, and none of the clenbuterol-treated mice died from heart conditions. Furthermore, clenbuterol did not modify the serum creatine kinase levels in both control and AR113Q mice (Supplementary Figure 3). β-agonists are known to increase the mass of skeletal muscle and reduce that of adipose tissue, an effect that is known as the “repartitioning effect”^22^. Therefore, we measured the mass of epididymal white adipose tissue (WAT). Clenbuterol decreased by 32% (p = 0.058) the mass of WAT in AR113Q mice. In AR113Q mice, muscles composed of mixed fibers, such as gastrocnemius, undergo a fiber-type switch towards oxidative metabolism^20^. Because β-agonist stimulation promotes a slow-to-fast fiber-type switch^22^, we hypothesized that clenbuterol attenuates the metabolic alterations detected in SBMA muscles. By NADH staining, we found that clenbuterol significantly (p = 0.001) decreases the number of oxidative fibers by 20% in the gastrocnemius muscle of 180-day-old AR113Q mice (Fig. 4B). Glycolytic fibers develop a more severe atrophy compared to oxidative fibers in AR113Q mice^20^. Clenbuterol increased by 5% and 11% the mean myofiber cross-sectional area (CSA) of oxidative and glycolytic fibers, respectively (Fig. 4C). Interestingly, clenbuterol increased by 1.5-fold the levels of expression of S6 (Fig. 4D), which is consistent with the notion that β-agonist stimulation promotes protein synthesis in muscle^22^. Importantly, the effect of clenbuterol was associated with 80% reduction of polyglutamine-expanded AR in muscle (Fig. 4E). No effect of clenbuterol on the Akt pathway and AR levels was detected in the brainstem and spinal cord (Supplementary Figure 4A–C). These observations indicate that clenbuterol ameliorates muscle pathology and decreases the accumulation of the disease protein in SBMA knock-in mice.

### Clenbuterol reduces the toxicity of polyglutamine-expanded AR in myotubes derived from SBMA patients

**Figure 5:**
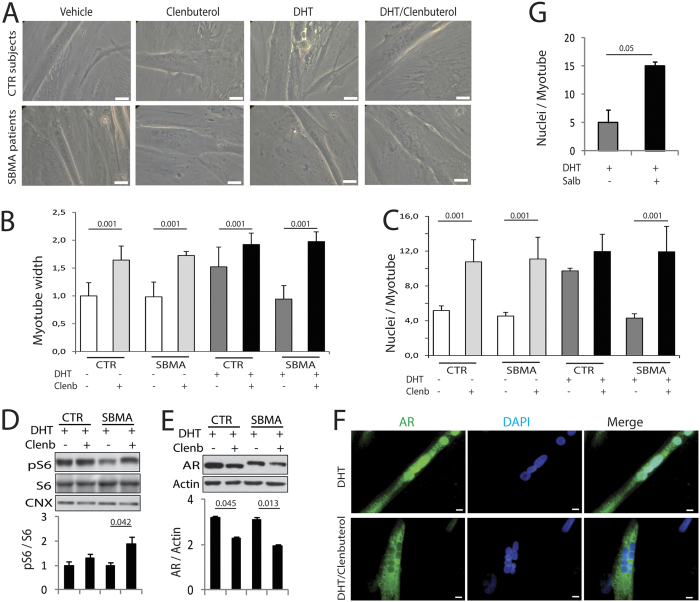
Clenbuterol attenuates the toxicity of polyglutamine-expanded AR in SBMA patient-derived myotubes. (**A**) Representative images of myotubes derived from SBMA patients and age-matched normal subjects (CTR) treated with vehicle, DHT (10 nM), and clenbuterol (Clenb, 10 μM) for 10 DIV. Bar, 20 μm. (**B**) Analysis of the width of myotubes derived from CTR subjects and SBMA patients treated as in (**A**). Graph, mean ± SEM, n = 3 CTR subjects, 4 SBMA patients. Two-way ANOVA + SNK. (**C**) Analysis of the number of nuclei/myotube in myotubes derived from CTR subjects and SBMA patients treated as in (**A**). Graph, mean ± SEM, n = 3 CTR subjects, 5 SBMA patients. Two-way ANOVA + SNK. (**D**,**E**) Western blotting analysis of phosphorylated and total S6 and total AR levels in myotubes obtained from CTR subjects and SBMA patients treated as in (**A**). AR, pS6, and S6 were detected with specific antibodies, and calnexin (CNX) and actin were used as loading control. Graph, mean ± SEM, n = 3 CTR subjects and SBMA patients. Two-way ANOVA + SNK. (**F**) Immunofluorescence analysis of AR in myotubes derived from SBMA patients treated as in (**A**). AR was detected with a specific antibody and nuclei with DAPI. Shown are representative images of myotubes derived from 3 SBMA patients. Bar, 10 μm. (**G**) Analysis of the number of nuclei/myotube in myotubes derived from CTR subjects and SBMA patients treated with DHT (10 nM) and either vehicle or salbutamol (Salb, 1 μM) for 10 DIV. Graph, mean ± SEM, n = 3 CTR subjects, 3 SBMA patients. Student’s t-test.

To determine whether clenbuterol modifies the toxicity of endogenous polyglutamine-expanded AR in myotubes derived from SBMA patients, we cultured primary myoblasts obtained from the quadriceps femoris muscle of SBMA patients and healthy individuals (Fig. 5A). Myoblasts were differentiated to myotubes for 10 DIV in the presence and absence of DHT and clenbuterol. Different from the C2C12 cells, in the absence of DHT clenbuterol increased the size of both control and SBMA myotubes by 1.6-fold (Fig. 5B). DHT treatment increased the size of control myotubes by 1.5-fold and had no effect on the SBMA myotubes, as previously shown by us^19^. In the presence of DHT clenbuterol increased the size of control and SBMA myotubes by 1.3-fold and 2.1-fold, respectively. Expression of polyglutamine-expanded AR reduces the fusion index of myotubes, thereby leading to altered myotube maturation^19^. In the absence of androgens clenbuterol increased the number of nuclei/myotube by 2.1-fold and 2.4-fold in control and SBMA myotubes, respectively (Fig. 5C). DHT increased by 1.9-fold the number of nuclei/myotube in control cells, and had no effect in SBMA myotubes, as previously reported by our group^19^. In the presence of DHT clenbuterol increased the number of nuclei/myotube of control and SBMA myotubes by 1.2- and 2.8-fold, respectively. Clenbuterol increased the phosphorylation of S6, suggesting that it promotes protein synthesis (Fig. 5D). Moreover, clenbuterol significantly reduced the accumulation of normal and polyglutamine-expanded AR by 29% and 37%, respectively (Fig. 5E). Accumulation of polyglutamine-expanded AR was increased in the nucleus of SBMA myotubes, as we have previously shown^19^, and decreased by clenbuterol (Fig. 5F). Finally, we tested the efficacy of a Food and Drug Administration (FDA)-approved β-agonist, salbutamol, in our SBMA cells (Fig. 5G). Salbutamol significantly increased the number of nuclei/myotube in the DHT-treated cells. These observations indicate that β-agonist stimulation reduces the accumulation of polyglutamine-expanded AR and promotes hypertrophy and differentiation of SBMA human primary myotubes.

## Discussion

Here we present evidence that β-adrenergic stimulation with clenbuterol protects C2C12 myotubes, knock-in mice, and patient-derived primary myotubes from the toxicity of polyglutamine-expanded AR. Clenbuterol activated the PI3K/AKT/mTOR signaling pathway in SBMA myotubes, and reduced the accumulation of the disease protein. Our preclinical study in mice showed that chronic administration of clenbuterol starting at disease onset reduces atrophy and metabolic alterations in muscle, ameliorates motor function, and extends survival. Based on these results and on a pilot trial that we carried out with clenbuterol on SBMA patients^29^, we propose β-agonist stimulation as a novel therapeutic approach for SBMA patients.

β-adrenergic stimulation has been shown to promote skeletal muscle hypertrophy and reduce muscle atrophy in several pathological conditions, such as cachexia, sepsis, and neuromuscular diseases^22^. Here we provide evidence that β-adrenergic stimulation is a new approach to counteract muscle wasting in SBMA. Clenbuterol ameliorated the phenotype of myotube cells derived from C2C12 myoblasts and SBMA patients. *In vivo*, clenbuterol increased muscle mass and force-producing capacity, improved motor function and extended the survival of AR113Q mice. We have previously shown that polyglutamine expansion in the AR causes a glycolytic-to-oxidative metabolic switch in the muscle of SBMA knock-in mice and patients that was associated with decreased expression of glycolytic genes, enhanced expression of lipid genes and lipid turnover, and increased expression of peroxisome proliferator-activated receptor gamma coactivator 1 alpha (PGC1α)^20^. Here, we found that clenbuterol attenuates the metabolic alterations observed in the muscle of SBMA mice. Notably, clenbuterol treatment increased force production in AR113Q mice. Because fast-glycolytic fibers produce more force than slow-oxidative fibers, the increase in the number of glycolytic fibers induced by clenbuterol may account for the effect on muscle force. In SBMA muscle, glycolytic fibers are more severely affected than oxidative fibers^20^. β-adrenoceptor agonists have been reported to exert greater effects on fast-twitch glycolytic fibers with respect to slow-twitch oxidative fibers^44^. Consistent with these observations, the effect of clenbuterol on the atrophy of glycolytic fibers was higher compared to that observed on the oxidative fibers. Slow-oxidative fibers use fatty acids as major energy source for oxidative phosphorylation. We have recently shown that the metabolic alterations detected in glycolytic muscles of AR113Q mice can be attenuated by feeding the mice a high-fat diet^20^. Different from clenbuterol, the high-fat diet ameliorated the size of oxidative, but not glycolytic fibers. Furthermore, the high-fat diet was administered starting from 40 days of age, when the mice started to show metabolic alterations. Here we show that chronic treatment of the mice with clenbuterol administered when the mice start to show body weight loss and muscle atrophy (13 weeks of age) is effective in reducing the metabolic alterations detected in SBMA knock-in mice.

Clenbuterol may also act in tissues other than muscle to ameliorate SBMA phenotype. β-agonist stimulation promotes lipolysis leading to increased release of free fatty acids^45^. Therefore, it is possible that clenbuterol ameliorates SBMA muscle pathology by promoting lipolysis and the release of free fatty acids from adipocytes. Indeed, clenbuterol treatment resulted in a reduction in WAT mass concomitant to an increase in skeletal muscle mass. This evidence suggests that β-agonist stimulation acts not only on the skeletal muscle, but also on the adipose tissue to attenuate muscle atrophy and ameliorate the phenotype of SBMA mice. Future analyses will establish which cellular pathways clenbuterol activates in WAT to promote lipolysis in the SBMA muscle. Moreover, based on the effect that clenbuterol has on WAT in SBMA mice, it will be important to determine what is the mechanism through which clenbuterol affects the adipose tissue and lipid metabolism in light of a future clinical trial in SBMA patients. In addition, clenbuterol may act on the central nervous system to modify SBMA phenotype. We did not observe changes in the activation of the Akt pathway and in the levels of expression of AR in the spinal cord and brainstem. Yet, analysis of muscle force elicited by presynaptic stimulation suggests that clenbuterol may exert beneficial effects on motor neurons. Indeed, clenbuterol has previously been shown to increase peripheral nerve regeneration after sciatic nerve transection^46^, and to reduce motor neuron abnormalities in motor neuron degeneration (mnd) mice^47^. Clenbuterol exerts trophic effects on motor neurons, which are sensitive to changes in the generation and release of neurotrophins, such as brain-derived neurotrophic factor (BDNF). Notably, BDNF levels are decreased in SBMA muscle^48^. BDNF has been shown to promote local protein synthesis to improve synaptic strength and function and to ameliorate axonal transport defects in SBMA^49^. Therefore, clenbuterol may act on motor neurons by increasing the release from muscle of neurotrophins, such as BDNF, which can in turn act on the innervating motor neuron to ameliorate SBMA phenotype.

Two neurotransmitters, the catecholamines adrenaline and noradrenaline, mediate the effects of the autonomic sympathetic nervous system through binding to α-adrenoceptors and β-adrenoceptors^22^. In skeletal muscle, the most abundant adrenoceptors are the β_2_-subtypes. Adrenoceptors induce muscle hypertrophy by activating the PI3K/Akt and mTOR pathways, thus increasing the rate of protein synthesis. We found that clenbuterol increases the phosphorylation of Akt and S6, suggesting that it stimulates new protein synthesis in SBMA muscle. This effect was specific to muscle, as no changes in the phosphorylation status of S6 were detected in the spinal cord and brainstem of SBMA mice. We have previously reported that the mTOR pathway is activated in SBMA muscle and restored to normal levels by a high-fat diet^20^. Opposite to the effects of high-fat diet, clenbuterol further enhanced the activation of this pathway. The beneficial effects of clenbuterol in SBMA muscle may involve the enhancement of the rate of new protein synthesis, which is mediated by the mTOR pathway. In addition, clenbuterol may act on different pathways that work synergistically with the mTOR pathway to induce muscle hypertrophy, such as the AC/PKA pathway. In addition to the key role in the maintenance of muscle homeostasis^30^, the PI3K/Akt pathway also has specific effects in SBMA and other polyglutamine diseases^50^. Akt phosphorylates normal and polyglutamine-expanded AR^32^,^33^,^51^. Activation of the PI3K/Akt pathway by the insulin-like growth factor 1 (IGF-1) in the skeletal muscle of SBMA transgenic mice resulted in phosphorylation of polyglutamine-expanded AR and proteasome-mediated AR degradation^31^,^34^. Here we found that clenbuterol activates the PI3K/Akt pathway and reduces the accumulation of normal and polyglutamine-expanded AR, suggesting that at least in part its hypertrophic effect is mediated by this signaling pathway, which promotes the clearance of the disease protein via the ubiquitin-proteasome system^9^.

β-agonist stimulation promotes muscle hypertrophy through activation of the AC/PKA pathways^22^. Activation of AC increases the production of cyclic adenosine monophosphate, which in turn activates PKA. PKA activation in muscle results in phosphorylation of specialized calcium release channels (RyRs), an event that increases the open probability of the channels leading to release of calcium from the sarcoplasmic reticulum and potentiation of muscle contraction^52^. A key effector of PKA is CREB. PKA-mediated CREB activation in muscle results in upregulation of transcription of several genes, such as the orphan nuclear receptors, NOR-1 and nur-77, which inhibit the expression of myostatin, a negative regulator of muscle mass^53^. In addition, CREB regulates the expression of myogenic factors, such as paired bow 3 (Pax3), myogenic differentiation 1 (MyoD), and myogenic factor 5 (Myf5)^54^, and induces the expression of salt-inducible kinase 1 (SIK1), which results in phosphorylation-dependent nuclear export of histone deacetylase 5 (HDAC5) and activation of myocyte enhancer factor-2 (Mef2)^55^. Clenbuterol treatment increased the phosphorylation of CREB in cultured SBMA myotubes. Even if the effect of clenbuterol was not significant, it is possible that the activation of the AC/PKA pathway also contributes to the beneficial effects of clenbuterol in SBMA muscle. Interestingly, β-agonist-induced PKA activation stimulates lipolysis through phosphorylation of hormone-sensitive lipase and perilipin A, which in turn increases the release of fatty acids from the adipose tissue^45^. We found that clenbuterol decreases the mass of WAT, suggesting that mobilization of fatty acids from adipose tissue may contribute to provide free lipids used by glycolytic muscles that have undergone metabolic shift towards oxidative phosphorylation.

Our results have clinical relevance for SBMA. Together with our pilot trial on SBMA patients^29^, our preclinical study provides further evidence that the use of clenbuterol is a valuable strategy for SBMA patient therapy. The efficacy of β-agonists has been proven in other animal models of motor neuron diseases, including ALS^24^,^56^, and mnd mice^47^. β-agonists, such as clenbuterol and terbutaline, have been shown to delay disease onset, ameliorate motor function and extend life span in transgenic mice overexpressing mutant superoxide dismutase 1 (SOD1)^24^, and Zebrafish overexpressing mutant TAR DNA-binding protein 43 (TDP-43) modeling ALS^27^. Interestingly, in the mutant SOD1-linked ALS model, the effect of clenbuterol was more pronounced in female compared to male mice. β-agonists have been used in clinical trials in support of patients suffering from motor neuron diseases. Clenbuterol ameliorated some aspects of disease in a pilot trial carried out on ALS patients^57^. Notably, β-agonist stimulation increased the expression of survival of motor neuron 2 (smn2), suggesting that, in addition to having an indirect effect on skeletal muscle atrophy, activation of β-adrenergic signaling ameliorates spinal muscular atrophy (SMA) phenotype by directly increasing the levels of expression of smn2^58^,^59^. In a pilot trial carried out on SBMA patients, we have previously shown that clenbuterol leads to a significant and sustained increase in specific functional measures, including 6-minute-walk-distance (6MWD) and forced vital capacity, between the baseline and the 12-month assessments^29^. These results are particularly encouraging since the natural history of SBMA is characterized by a drop of 6MWD at a rate of 11.3% per year. These observations indicate that β-agonist stimulation has therapeutic potential in motor neuron diseases and, more specifically, in SBMA. However, clenbuterol is not FDA-approved. Interestingly, salbutamol, an FDA-approved β-agonist, improved forced vital capacity, lean body mass, and muscle strength in clinical trials carried out on SMA patients^60^,^61^, and ameliorated the phenotype of myotubes derived from SBMA patients, thereby highlighting its role as an effective β-agonist for motor neuron diseases.

The use of β-agonists may be associated with side effects that may limit their translation into the clinic. This aspect is critical when prolonged treatment is required, as is the case of chronic neurodegenerative diseases, such as ALS and SBMA. Common side effects of β-agonists are nausea, headache, insomnia, muscle tremor and cramps, which are symptoms that occur early in treatment and are reduced with continued use of the drug^29^. More importantly, β-agonist stimulation can exert toxic effects on heart, leading to palpitation, tachycardia, and cardiac hypertrophy^62^. This aspect is important, as SBMA patients may suffer from Brugada syndrome^63^. However, we did not detect any sign of cardiac muscle hypertrophy and heart rhythm complications in SBMA mice and patients treated with clenbuterol^29^. Another concern about the use of β-agonists is CK elevation in both animals and humans^29^,^64^,^65^. Although CK levels did not change in the clenbuterol-treated mice compared to the vehicle-treated animals, CK levels were increased in patients treated with clenbuterol, thus requiring CK monitoring in long term treatment with clenbuterol. The significance of this finding is unclear but, at least in SBMA, we speculate that anabolic stimulation may cause a collapse of some hypertrophic fibers, rather than major muscle damage^29^.

In conclusion, our preclinical study shows that β-agonist stimulation ameliorates the phenotype of SBMA mice and patient-derived myotubes. Our findings provide a solid rationale for a future phase II clinical trial for testing efficacy and safety of clenbuterol in SBMA.

## Additional Information

**How to cite this article:** Milioto, C. *et al*. Beta-agonist stimulation ameliorates the phenotype of spinal and bulbar muscular atrophy mice and patient-derived myotubes. *Sci. Rep.* **7**, 41046; doi: 10.1038/srep41046 (2017).

**Publisher's note:** Springer Nature remains neutral with regard to jurisdictional claims in published maps and institutional affiliations.

## Supplementary Material

Supplementary Material
