# Supplementary material for: Beta-agonist stimulation ameliorates the phenotype of spinal and bulbar muscular atrophy mice and patient-derived myotubes
Source: Sci Rep. 2017 Jan 24;7:41046. doi: 10.1038/srep41046 (PMC5259768; doi:10.1038/srep41046)
Supplement: Supplementary Material [file srep41046-s1.pdf]

Beta-agonist stimulation ameliorates the phenotype of spinal and bulbar muscular atrophy mice and patient-derived myotubes

Carmelo Milioto[1,2,3], Adriana Malena[4], Eleonora Maino[1], Maria J. Polanco[1], Caterina Marchioretti[1], Doriana Borgia[4], Marcelo Gomes Pereira[5], Bert Blaauw[5], Andrew Lieberman[6], Roberta Venturini[7], Mario Plebani[7], Fabio Sambataro[8], Lodovica Vergani[4], Elena Pegoraro[4], Gianni Sorarù[4]*, Maria Pennuto[1]*

[1] Dulbecco Telethon Institute, Centre for Integrative Biology, University of Trento, 38123 Trento, Italy

[2] Department of Neuroscience and Brain Technologies, Istituto Italiano di Tecnologia, 16163 Genova, Italy

[3]Dipartimento di Medicina Sperimentale, University of Genova, 16100 Genova, Italy

[4]Department of Neurosciences, University of Padova, 35100 Padova, Italy

[5]Venetian Institute of Molecular Medicine, Department of Biomedical Science, University of Padova, 35100 Padova, Italy

[6]Department of Pathology, University of Michigan Medical School, Ann Arbor, MI 48109, USA

[7] Department of Laboratory Medicine, University Hospital of Padova, 35100 Padova, Italy.

[8] Department of Experimental & Clinical Medical Sciences (DISM), University of Udine, 33100 Udine, Italy

* Corresponding authors:

Gianni Sorarù, MD, PhD
Motor Neuron Disease Clinic
Department of Neurosciences, University of Padova
Via Giustiniani 2, 35128 Padova, Italy
Phone +39 0498216600
Fax +390498751770
Gianni.soraru@unipd.it

Maria Pennuto, PhD
Dulbecco Telethon Lab of Neurodegenerative Diseases
CIBIO, University of Trento
Via Sommarive 9, 38123 Trento, Italy
Phone +39 0461 285215
Fax: +39 0461 283937
MPennuto@Dti.Telethon.it

Supplementary Figure 1

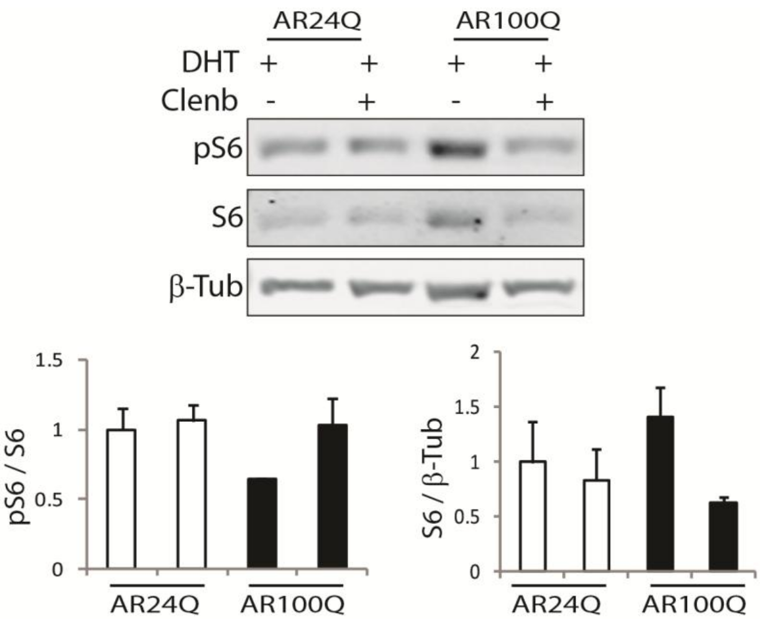

Supplementary Figure 1. Clenbuterol increases S6 phosphorylation in SBMA C2C12 cells.

Western blotting analysis of C2C12 myotubes expressing AR24Q and AR100Q and treated with DHT (10 nM) and clenbuterol (Clenb, 10 μM) for 14 DIV. Phosphorylated and total S6 were detected with specific antibodies, and beta-Tubulin (β-Tub) was used as loading control. Graph, mean ± SEM, n = 3 independent experiments. Two-way ANOVA + Newman-Keuls test.

Supplementary Figure 2

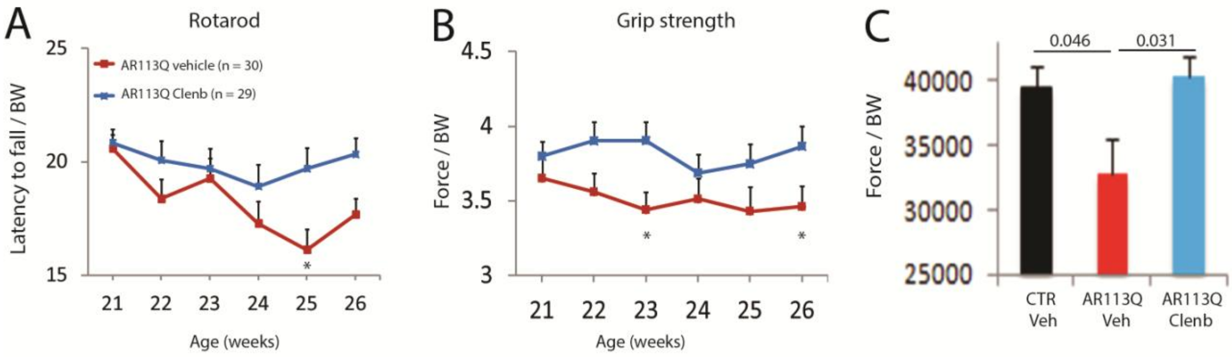

Supplementary Figure 2. Clenbuterol ameliorates the motor function and muscle force of AR113Q mice.

A) Rotarod analysis of motor coordination in CTR and AR113Q mice treated with vehicle and clenbuterol (Clenb, 2 mg/kg) normalized to body weight (BW). Graph, mean ± SEM. Week 25: * p = 0.05 AR113Q-vehicle vs AR113Q-clenbuterol.

B) Grip strength analysis of CTR and AR113Q mice normalized to body weight (BW). Graph, mean ± SEM. Week 23: * p = 0.01 AR113Q-vehicle vs AR113Q-clenbuterol. Week 26: * p = 0.041 AR113Q-vehicle vs AR113Q-clenbuterol.

C) *In vivo* force generation of gastrocnemius muscle measured in live 180-day-old AR113Q and CTR mice and normalized to body weight (BW). Graph, mean ± SEM, n = 4 mice.

Supplementary Figure 3

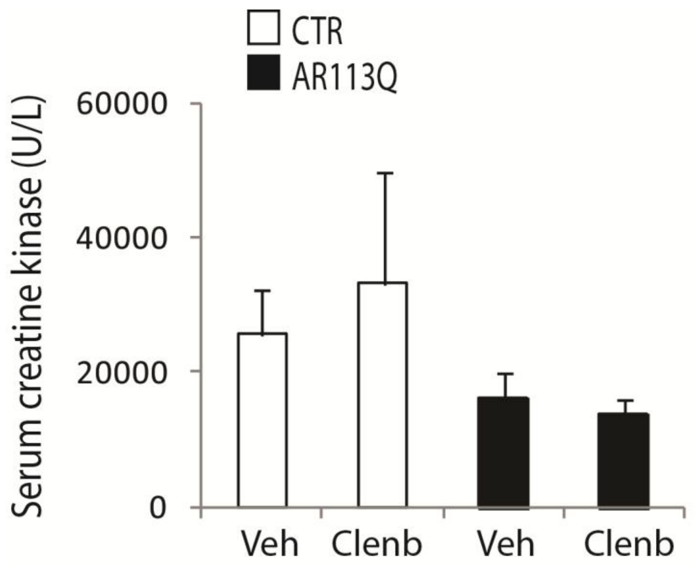

Supplementary Figure 3. Clenbuterol does not modify the serum levels of creatine kinase. The levels of creatine kinase (CK) were measured in the serum of 180-day-old control (CTR, wild type) and AR113Q mice treated with vehicle and clenbuterol (Clenb, 2 mg/kg). Serum CK levels were not modified by treatment. Graph, mean ± SEM, n = 6 CTR-vehicle, 6 CTR-clenbuterol, 8 AR113Q-vehicle, and 6 AR113Q-clenbuterol mice. Two-way ANOVA + Newman-Keuls test.

Supplementary Figure 4

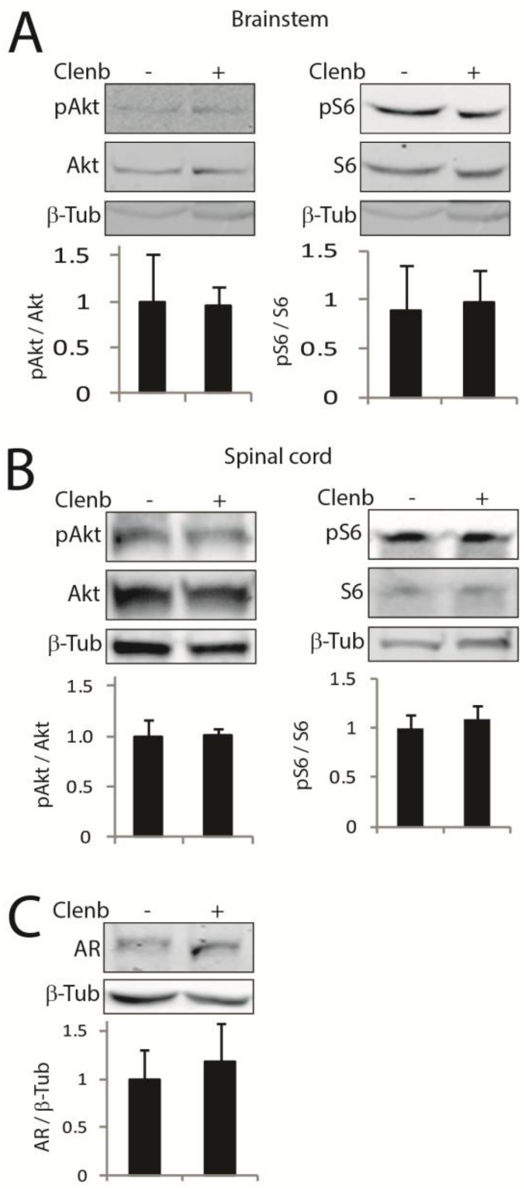

Supplementary Figure 4. Clenbuterol does not modify the Akt pathway and AR levels in the brainstem and spinal cord of SBMA mice.

A-B) Western blotting analysis of phosphorylated and total Akt and S6 levels in the brainstem and spinal cord of 180-day-old AR113Q mice treated with vehicle and clenbuterol (Clenb, 2 mg/kg). Phosphorylated and total Akt and S6 were detected with specific antibodies, and beta-Tubulin (β-Tub) was used as loading control. Graph, mean ± SEM, n = 6 mice for each group.

C) Western blotting analysis of AR levels in the spinal cord of 180-day-old AR113Q mice treated with vehicle and clenbuterol (Clenb, 2 mg/kg). AR was detected with a specific antibody and beta-Tubulin (β-Tub) was used as loading control. Graph, mean ± SEM, n = 6 mice for each group.

Supplementary Figure 5

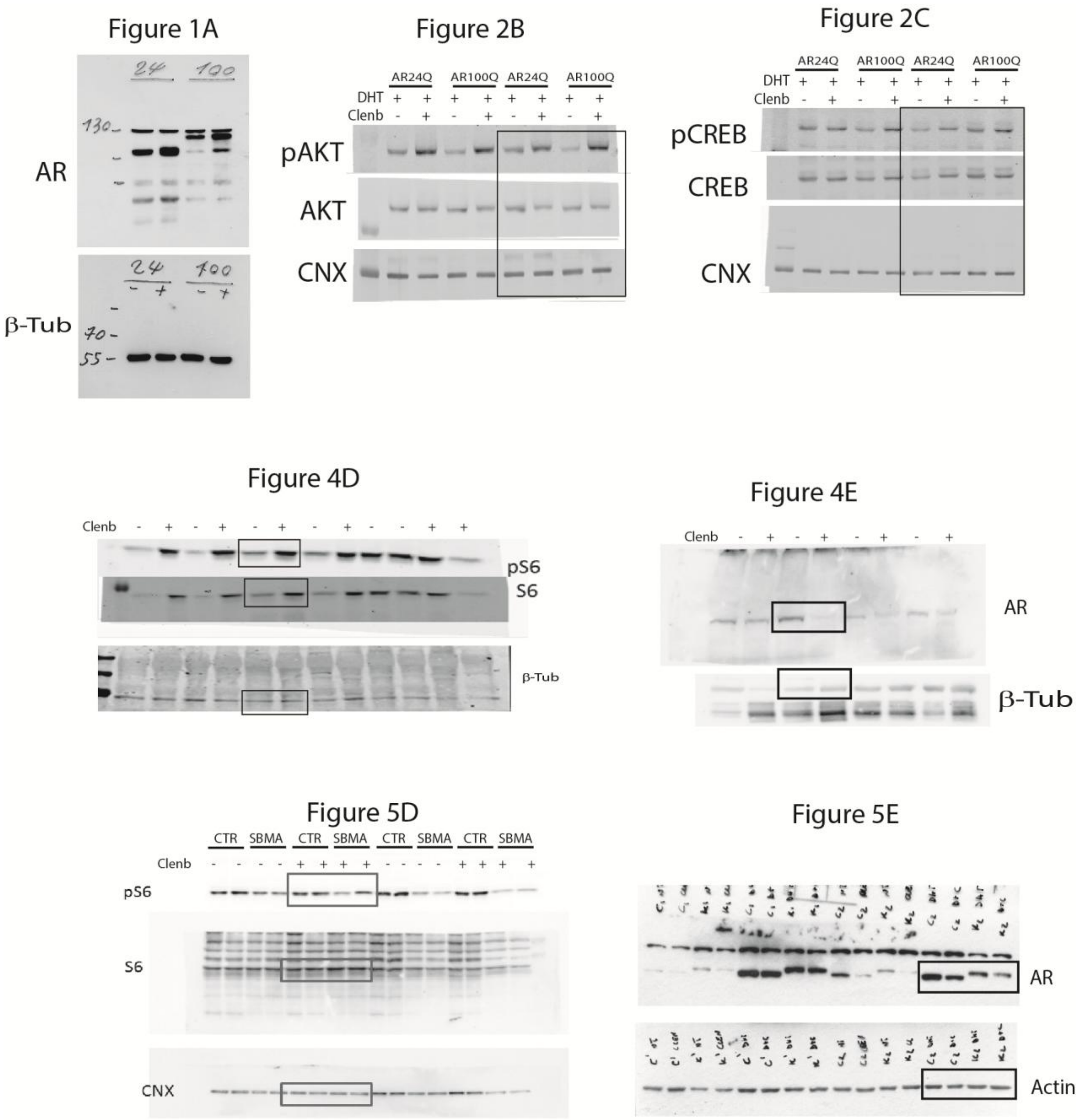

Supplementary Figure 5. Uncropped Western blotting images.
